# Orally delivered polycurcumin responsive to bacterial reduction for targeted therapy of inflammatory bowel disease

**DOI:** 10.1080/10717544.2016.1245367

**Published:** 2017-02-03

**Authors:** Hongzhi Qiao, Dong Fang, Jing Chen, Yuan Sun, Chen Kang, Liuqing Di, Junsong Li, Zhipeng Chen, Jun Chen, Yahan Gao

**Affiliations:** 1 School of Pharmacy, Jiangsu Engineering Research Center for Efficient Delivery System of TCM, Nanjing University of Chinese Medicine, Nanjing, China,; 2 Department of Chemistry and Biochemistry and College of Pharmacy, The Ohio State University, Columbus, OH, USA,; 3 Division of Pharmacology, College of Pharmacy, The Ohio State University, Columbus, OH, USA, and; 4 Department of Pharmaceutics, State Key Laboratory of Natural Medicines, China Pharmaceutical University, Nanjing, China

**Keywords:** Curcumin polymer, colon-targeting, bacterial reduction, inflammatory bowel disease, oral administration

## Abstract

Inflammatory bowel disease (IBD) such as Crohn’s disease and ulcerative colitis is a chronic autoimmune disease affecting nearly five million people worldwide. Among all drug delivery system, oral administration is the most preferable route for colon-specific targeting and the treatment of IBD. Herein, an amphiphilic curcumin polymer (PCur) composed of hydrophilic poly(ethylene glycol) (PEG) and hydrophobic curcumin (Cur) linked by disulfide bond was synthesized and characterized. The sufficient solubility, nano-scaled size and close to the neutral surface potential of PCur lead to preferential accumulation of the active drug in the inflamed regions of the gut. Moreover, PCur showed limited drug release and enhanced robustness under the physiological pH of the gastrointestinal tract (GIT), and a significantly elevated release was observed when responding to a bacterial reduction in the colon. Furthermore, cellular studies confirmed PCur had low cytotoxicity and increased transmembrane permeability, resulting in improved oral bioavailability evidenced by *in vivo* pharmacokinetics of rats. Finally, with DSS-induced murine model of IBD, we demonstrated that orally administered PCur ameliorated the inflammatory progression in the colon and could protect mice from IBD. In conclusion, it is illustrated that the developed PCur conjugate could potentially be employed as a colon-specific candidate for IBD treatment.

## Introduction

Inflammatory bowel disease (IBD), comprising Crohn’s disease (CD) and ulcerative colitis (UC), is described as chronic but relapsing inflammatory disorders of the gastrointestinal tract (GIT) (Abraham & Cho, 2009; Khor et al., 2011). Most common symptoms for patients with IBD are abdominal pain, diarrhea, rectal bleeding and weight loss. CD tends to exist in any part of GIT and is characterized by transmural inflammation which affects the whole bowel wall and reaches the serosal layer. On the contrary, UC is typically confined to the superficial regions of colon and rectum, disturbing only the mucosal layer (Abraham & Cho, 2009). Anti-inflammatory drugs, immunosuppressants and TNF blockers are prescribed clinically for the treatment of IBD (Nielsen & Ainsworth, 2013). However, the high cost and adverse effects of these drugs in concert with their nonspecific efficacy after administration necessitate the exploration of other therapeutic options.

Curcumin (Cur), a natural hydrophobic polyphenol derived from the rhizomes of turmeric, *Curcuma longa*, is holding considerable attention for IBD treatment in terms of low cost, reliable sources and excellent anti-inflammatory activity (Sreedhar et al., 2016). A substantial amount of clinical trials has reported that Cur showed better efficacy over placebo in the alleviation of IBD (Lahiff & Moss, 2011). However, the further clinical application of Cur for the treatment of digestive disorders has been largely hindered by its poor aqueous solubility and bioavailability (Dulbecco & Savarino, 2013). Therefore, there is an urgent need to develop an effective delivery strategy toward IBD therapy with satisfactory partition coefficients, specific targeting to inflamed colonic tissue, enhanced therapeutic efficacy and minimal side effects.

Nano-sized drug delivery systems have drawn significant attention for IBD treatment due to their preferential accumulation in the inflamed regions of the gut (Youshia & Lamprecht, 2016). Nanoparticle with the size of ∼100 nm was found to provide the highest deposition in inflamed tissue (Lamprecht et al., 2001). Additionally, particle surface plays a crucial role in the interaction with the mucus layer and epithelial cells (Lautenschlager et al., 2013). Nonionic surfactants have been verified to be more favorable for IBD targeting compared to anionic and cationic surfactants (Lautenschlager et al., 2013). Furthermore, a number of techniques such as pH, time, or pressure respondent devices were employed in the delivery strategies for specific targeting to the colon (Wilson et al., 2010; Beloqui et al., 2013). Thereinto, the colonic bacterial azo reduction has a long history of exploitation for the specific delivery of drugs to the colon (Sinha & Kumria, 2003). The earliest example is set for prodrug sulfasalazine, which releases the active drug 5-aminosalicylic acid (5-ASA) upon reduction, and was extensively used for the treatment of IBD. To date, very little study has been reported to investigate polymeric systems with other reducible groups (e.g. disulfide bond) for colon targeted drug delivery, despite the fact that a handful of small molecular compounds containing disulfide bonds have been reported to acquire the ability to be reduced in the gut (Saphier et al., 2012).

In this study, we synthesized an amphiphilic polycurcumin (PCur) by conjugating hydrophilic poly(ethylene glycol) (PEG) and hydrophobic Cur with disulfide bonds, which is designed for responding to the bacterial reducing environment in the colon. Nano-scaled size and neutral charged surface will endow PCur with a preferential deposition in inflamed tissue, while improved partition coefficient will facilitate transmembrane transport and increase oral bioavailability. Furthermore, the evaluation of therapeutic efficacy was conducted on the murine IBD model through oral administration of colon-targeted reduction-sensitive PCur conjugate.

## Materials and methods

### Materials

Polyethylene glycol (PEG, Mn ∼2000) was purchased from Sigma-Aldrich (Shanghai, China). 3,3′-Dithiodipropionic acid, 1-ethyl-3-(3-dimethylaminopropyl) carbodiimide (EDC) and 4-dimethylaminopyridine (DMAP) were supplied by Aladdin-Reagent Co., Ltd. (Shanghai, China) and used as received. Curcumin (high purity, Cur) and chloramphenicol were obtained from Adamas-beta (Shanghai, China). Anhydrous tetrahydrofuran (THF) was provided by Sinopharm Chemical Reagent Co., Ltd. (Shanghai, China) and distilled prior to use. Dextran sulfate sodium (DSS, 35 000 Da) was purchased from MP Biomedicals Inc. (Santa Ana, CA). Purified deionized water was prepared by a Milli-Q® Plus System (Millipore Co., Billerica, MA). All other reagents and chemicals were of analytical grade and used without further treatment.

Caco-2 cells were obtained from the cell bank of Chinese Academy of Sciences (Beijing, China). The cells were cultured in DMEM with 10% (v/v) FBS, 1% (v/v) nonessential amino acids, 100 U/mL penicillin and 100 mg/mL streptomycin in an incubator (Thermo Scientific, Waltham, MA) at 37 °C under an atmosphere of 5% CO_2_ and 90% relative humidity. Cells were subcultivated approximately every five days (at 80% confluence) using trypsin-EDTA at a split ratio of 1:10.

Sprague–Dawley (SD) rats (180–220 g) and female C57BL/6 mice (eight weeks, 18–20 g) were purchased from the Laboratory Animal Center at Yangzhou University. All the animals were pathogen free and allowed to access to food and water freely. Animal experiments were carried out by the Guidelines for Animal Experimentation of Nanjing University of Chinese Medicine (Nanjing, China) and the protocol was approved by the Animal Ethics Committee of this institution.

### Synthesis of PEG-ss-curcumin (PCur) conjugate

PCur was prepared by condensation polymerization reaction as previously described with minor modifications (Tang et al., 2010). 1.10 g Cur, 1.25 g 3,3′-dithiodipropionic acid, 6.02 g PEG, 2.42 g EDC and 0.14 g DMAP were dissolved in 100 mL anhydrous THF. The mixture was stirred at room temperature for 24 h, and an excess of cold anhydrous ether was added twice to precipitate and isolate the crude product. The product was dried under vacuum, and dialyzed against water for 36 h and lyophilized to produce yellow powder 6.69 g (yield 80%). ^1^H NMR (DMSO-*d*_6_, ppm): 7.6 (br, C*H*_b_=CH_c_), 6.8–7.3 (br, C_6_H_3_), 6.6 (br, CH_b_=C*H*_c_), 6.1 (br, C*H*_d_=C–OH), 4.4 (s, COOC*H*_2_CH_2_O), 3.8 (s, C*H*_3_OC_6_H_3_), 3.5–3.7 (br, OC*H*_2_CH_2_O), 3.3 (s, OCH_2_CH_2_OC*H*_3_), 2.7–3.0 (m, –C*H*_2_C*H*_2_–S–S–C*H*_2_C*H*_2_–). MALDI MS: 12 250.

### Characterization of PCur conjugate

^1^H NMR spectrum was performed on a Bruker Avance spectrometer (Bruker, Karlsruhe, Germany), operating at 300 MHz with DMSO-*d*_6_ as solvent and TMS as internal standard (IS). The integrations of the peaks from Cur aromatic protons (6.8–7.3 ppm) and PEG ethylene protons (3.5–3.7 ppm) were used to calculate the Cur content in the polymer. MALDI-MS was performed in positive mode on a Voyager Elite mass spectrometer with delayed extraction technology (PerSeptive Biosystems, Framingham, MA). Sinapinic acid was used as matrix, and samples were prepared in a 50:50 (v/v) mixture of 0.1% TFA/acetonitrile. A 1-pmol/L solution of bovine serum albumin was added as IS. FT-IR spectrum was recorded as a solid state on Tensor 37 FTIR Spectrometer (Bruker, Karlsruhe, Germany).

Differential scanning calorimeter (DSC) was conducted on a DSC-204 analyzer (Netzsch, Selb, Germany). Each sample was sealed in aluminum hermetic pans and heated from 40 °C to 240 °C at a heating rate of 10 °C/min under an atmosphere of nitrogen gas. Data analysis was performed using NETZSCH-Proteus software (Netzsch, Selb, Germany).

The particle size and zeta potential of PCur in water at the concentration of 10 mg/mL was measured using a dynamic light scattering (DLS) (Nano-ZS90, Malvern Instruments Ltd., Malvern, UK) at 25 °C and a scattering angle 90° after dilution of PCur with distilled water.

The surface morphology of PCur was observed using transmission electron microscopy (TEM, Hitachi H-7650, Tokyo, Japan). A drop of micellar solution was deposited on a carbon-coated lacey support copper grid, stained with 1% phosphotungstic acid followed by air dry and then examined under TEM.

Cur content in PCur was measured by fluorescence spectra (FluoroMax-4, HORIBA Jobin Yvon, Milano, Italy) at excitation of 423 nm. Drug-loading content (DLC) was defined as the percentage of the drug to the total weight of PCur.

### *In vitro* release study of PCur conjugate

To investigate the ability of PCur to respond to reducing environment, the *in vitro* release profiles of Cur from PCur were performed in different media: pH 7.4 phosphate buffered saline (PBS), pH 7.4 PBS with 10 μM GSH, and pH 7.4 PBS with 10 mM GSH. Moreover, rat cecal suspension from anaerobic cultures was prepared to evaluate the release of PCur in colonic reducing environment (Saphier et al., 2012). Sterilized cecal contents were also used as a control. General operation was summarized as follows: 10 mg of PCur was suspended in 1 mL dissolution solution, transferred into a 3500 MWCO dialysis bag, immersed in 50 mL release medium containing 0.1% (w/v) Tween 80 and gently shaken at 100 rpm in a water bath (37.0 ± 0.5 °C). At predetermined intervals, 5 mL dissolution sample was collected, and the concentration of Cur was measured by fluorescent spectrometry.

Effect of pH values on drug release was also evaluated in the following buffers: hydrochloric acid/potassium chloride (pH 1.2), acetic acid/sodium acetate (pH 6.5) and PBS (pH 7.4). Buffer selection was based on the normal variations of the pH in the GIT from the stomach (pH 1.5) and the proximal small intestine (pH 6.0–6.8) to the ileocecal region (pH 7.3) (Hua et al., 2015). One milliliter of sample (containing 1.2 mg of Cur) was placed in a dialysis bag, immersed into each media containing 0.1% (w/v) Tween 80 for drug release.

### *In vitro* stability testing of PCur conjugate

For the study of PCur stability, 10 mg/mL freshly prepared PCur was incubated at 37 °C with pH 1.2 hydrochloric acid/potassium chloride solution, pH 7.4 PBS and pH 7.4 PBS containing 10 mM GSH, respectively. A small portion of solution was collected at predetermined time points to measure the size distribution of PCur conjugate by DLS. The morphology change of PCur was visualized by TEM while incubating in pH 7.4 PBS containing 10 mM GSH for 12 h.

### Cytotoxicity study and LDH release assay

*In vitro* cytotoxicity of PCur and Cur solution was assessed in Caco-2 cells by MTT assay. Caco-2 cells were seeded at a density of 1 × 10^5^ cells/mL in 100 μL DMEM culture medium in a 96-well plate at 37 °C and 5% CO_2_. After 24 h of incubation, Caco-2 cells were exposed to Cur and PCur solution with different Cur concentrations for 2 h. Meanwhile, serum-free medium incubation without drug was utilized as a control. MTT solution (20 μL, 5 mg/mL in PBS) was then added to each well, and the cells were further incubated for 4 h. The supernatant was removed and 100 μL of DMSO was introduced to each well to dissolve formazan crystals. Optical density (OD) was measured with a microplate reader (Thermo Scientific, Waltham, MA) at 570 nm. The experiments were repeated at least four times. Cell viability (%) was calculated as the ratio of OD in test samples to OD in control samples.

A lactate dehydrogenase (LDH) assay kit (Beyotime, Haimen, China) was used to determine the ability of PCur to destroy cell membranes. Briefly, Caco-2 cells seeded in a 96-well plate were incubated with Cur solution and PCur at 25 μg/mL concentration of Cur for 2 h, and serum-free medium was used as a control. After 1 h of incubation, 20 μL LDH release medium was added, followed by 1 h of co-incubation. One hundred and twenty microliter supernatant was transferred to a new plate at a determined time interval, followed by the addition of 60 μL LDH test medium and incubated for 30 min with gentle shaking. Finally, the absorption was measured at 490 nm on a microplate reader (Bio-Rad 500, Hercules, CA). The relative percentage (%) was calculated similarly as the above.

### Transcellular studies

Caco-2 cells were seeded at a density of 1 × 10^5^ cells per well on inserts with polycarbonate membranes (Transwell®, 12-well, 0.4 m pore size (Corning Inc., Corning, NY)). A 400 μL of culture medium was added to the apical (AP) side, and 600 μL was added to the basolateral (BL) side. The medium in both the AP and BL sides was changed every two days for the first week and every day thereafter. After 21 days of incubation, the integrity of the cell monolayers was evaluated by measuring transepithelial electrical resistance (TEER) values using a Millicell® electrical resistance system (Millipore, Billerica, MA). When the TEER value of inserts exceeded 350 cm^2^, indicating cell monolayers were integrated and tight junctions were intact, they were selected for further transport studies. For transcellular research, the culture medium in the AP and BL side was removed and changed by HBSS. Four hundred microliter test solution of either Cur or PCur, with equal concentrations of Cur (25 μg/mL), was added to the AP side. Six hundred microliter of fresh HBSS was added to the BL side. One hundred microliter HBSS was aspirated from the BL side and replaced with 37 °C fresh HBSS at 0.5, 1, 1.5 and 2 h after incubation. One hundred microliter of sample was mixed with 50 μL chloramphenicol (IS) and 50 μL acetonitrile, vortexed and centrifuged. The concentration of Cur in supernatant was determined by UPLC–MS/MS. At the end of the experiment, TEER values were assayed to estimate the integrity of the cell monolayers. The apparent permeability coefficient (*P*_app_) for Cur was calculated according to the following equation:

$$P_{\mathrm{app}}=(dQ/dt)/(A\times C_{0})$$

where the d*Q*/d*t* was the drug permeation rate, *A* was the surface area of polycarbonate membrane and *C*_0_ was the initial concentration of Cur in AP side.

### Pharmacokinetic study

Pharmacokinetics of PCur and Cur suspension were tested in SD rats, which were randomly divided into two study groups with six in each group. PCur and Cur suspension were given orally at a dose of 50 mg/kg of Cur. Three hundred microliter of blood samples were collected at 5, 10, 15, 30 and 45 min, 1, 2, 4, 8, 12 and 24 h post oral gavage and centrifuged at 12 000 rpm for 10 min. The supernatant plasma was collected and stored at −20 °C for UPLC–MS/MS analysis.

The concentration of Cur was determined by a Waters ACQUITY UPLC system using a Xevo Triple Quadrupole MS equipped with an electrospray ionization (ESI) interface (Waters Co., Milford, MA). One hundred microliter plasma was mixed with 50 μL chloramphenicol (IS) and 50 μL acetonitrile, followed by vortexing for 3 min, and centrifuged at 12 000 rpm for 10 min. The upper organic layers were evaporated, reconstituted in 100 μL mobile phase, and centrifuged for another 10 min at 12 000 rpm. An aliquot of 5 μL was injected into the UPLC–MS/MS for analysis. Pharmacokinetic parameters were calculated using DAS 2.1 software (BioGuider Co., Shanghai, China).

### *In vivo* distribution of PCur in intestinal tract of IBD mice

To study the distribution of PCur in intestine tract, female C57BL/6 mice (eight weeks, 18–20 g) receiving 3% (wt./vol) DSS were given a daily gavage of 200 μL PBS solution or PBS containing PCur (50 mg Cur per kg, *n* = 6). After seven days, each mouse without PCur treatment had lost about 10% of its body weight and had elevated fecal blood levels consistent with disease development. The total intestinal segments were removed, washed with cold PBS once, and the perfusate was collected for composition detection. Different intestinal segments including duodenum, jejunum, ileum, cecum and colon were isolated respectively, and then mucous and muscular layers were separated, homogenized and centrifuged at 12 000 rpm for 20 min. Finally, the supernatant was injected into HPLC for quantitative analysis of Cur.

### Pharmacodynamics of PCur in a murine DSS-induced IBD model

#### Experimental design

Animal experiments were performed in female C57BL/6 mice (eight weeks, 18–20 g). Colitis was induced by feeding mice with a 3% (wt./vol) DSS solution as drinking water for seven days. The daily evaluation of mice was based on changes in body weight and development of the clinical symptoms of colitis. Disease activity index (DAI) was calculated according to the parameter previously reported (Li et al., 2015). Starting on day zero, mice receiving DSS were given a daily gavage of PBS (200 μL) or a PBS solution (200 μL) containing one of the following: Cur suspension, PCur, or sulfasalazine (SSZ, as a positive control). The dosage of Cur was set at 50 mg/kg. Mice were sacrificed on day 7, and histological assessment of colonic inflammation was carried out by hematoxylin and eosin (H&E) staining of 5 μm colonic tissue sections, observed by microscopy (×20 magnification). The samples were scored for inflammatory infiltration (0–3), epithelial abnormality (0–3) and crypt damage (0–3) and then the values of the three variables were summed as an indicator of inflamed degree (Magro et al., 2013).

#### Myeloperoxidase (MPO) activity and malondialdehyde (MDA) content

Neutrophil infiltration into the colon can be quantified by measuring MPO activity. Briefly, a portion of the colon was homogenized in 1:20 (w/v) of 50 mM phosphate buffer (pH 6.0) containing 0.5% hexadecyltrimethyl ammonium bromide on ice using a homogenizer. The homogenate was then sonicated for 10 s, freeze-thawed three times, and centrifuged at 12 000 rpm for 15 min. The supernatant was then added to 1 mg/mL o-dianisidine hydrochloride and 0.0005% hydrogen peroxide, and the change in absorbance at 460 nm was measured.

Lipid peroxidation was evaluated by measuring the MDA content of the colon according to standard procedure protocol described in MDA Assay Kit (Beyotime, Haimen, China). In short, the colorimetric determination of MDA is based on the reaction of the reactive aldehyde with thiobarbituric acid at low pH (2–3) and temperature of 95 °C for 45 min. The pinkish resultant was extracted by n-butanol, and the absorbance was determined at 535 and 520 nm spectrophotometrically. The difference in OD between both wavelengths was used as a measure of colonic MDA content. The final value of MDA was represented as nmol/mg protein.

#### Determination of interleukin-6 (IL-6) and tumor necrosis factor-alpha (TNF-α) levels

The levels of IL-6 and TNF-α in homogenized colonic tissue were measured by quantitative enzyme-linked immunosorbent assay (ELISA) kits according to manufacturer’s instructions (R&D Systems Inc., Minneapolis, MN). The final values of IL-6 and TNF-α were expressed as pg/mg or ng/mg protein (Qiao et al., 2014).

### Statistical analysis

All experiments were conducted at least in triplicate; data were expressed as mean ± standard deviation (SD). Statistical analysis was performed using Sigma Stat software (Jandel Scientific Software, San Rafael, CA). Comparisons between groups were made using one-way ANOVA, followed by Student's *t*-test. Statistically significant difference was considered at *p* < 0.05.

## Results and discussion

### Synthesis and characterization of PEG-ss-curcumin (PCur) conjugate

**Figure 1.**
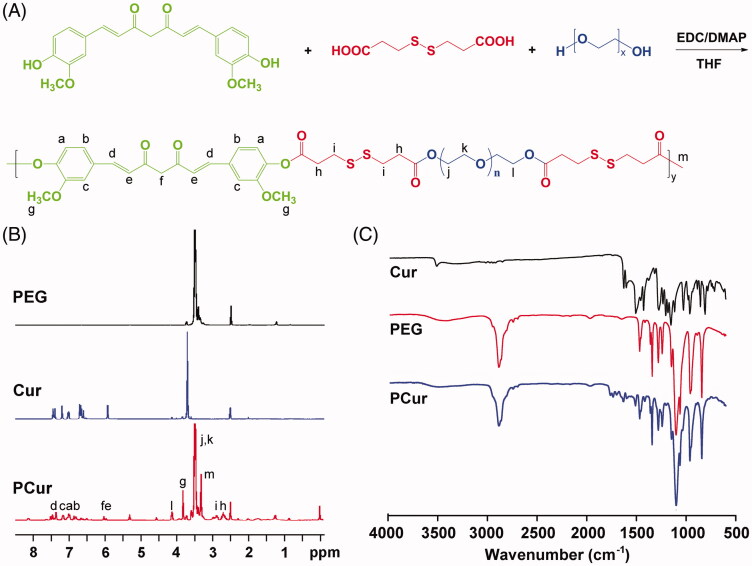
Synthetic scheme of PCur conjugate (A); representative ^1^H NMR (B) and FT-IR (C) spectra of original reactants (PEG and Cur) and the final product (PCur).

**Figure 2.**
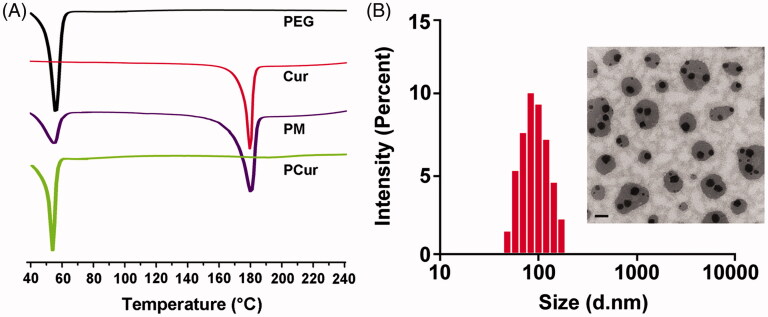
DSC thermograms of PEG, Cur, physical mixture (PM) and PCur conjugate (A); particle size distribution and morphology of PCur conjugate measured by DLS and TEM, respectively (B). Scale bar is 200 nm.

PCur conjugate was synthesized by condensation polymerization between Cur and PEG through the linker 3, 3′-dithiodipropionic acid (Figure 1A). Representative structural information of PEG and Cur incorporated in PCur were characterized by ^1^H NMR and FT-IR (Figure 1B and C), and the molecular weight was confirmed by MALDI mass spectrum. The loading content was calculated as 12.5% based on the ratio of the integrated area from the ^1^H NMR spectra of PCur, which was very close to the result determined by fluorescence spectra (∼14.6%). Solubility test revealed that the introduction of PEG remarkably increases the water solubility to as high as 1.6 mg/mL of Cur in PCur solution, where no obvious aggregation was detected (data not shown). Indeed, the solubility of the resulting PCur could be tailored by adjusting the Cur/PEG ratio. Incorporation of more PEG chains would lead to enhanced water-solubility while relatively decreased drug content for PCur. In Figure 2(A), results from DSC showed the endothermic peaks at 52.2 °C and 181.4 °C, attributing to melting of PEG and Cur, respectively. The characteristic peaks of Cur was not detected in PCur, but did appear in the physical mixture of Cur and PEG, which further confirmed the absence of free Cur in the PCur (Dandekar et al., 2010). DLS and TEM results (Figure 2B) showed PCur was well dispersed in a spherical shape with an average particle size of 134.4 ± 4.2 nm. Zeta potential was measured to be close to neutral (−3.3 ± 1.2 mV) attributed to nonionic features of PEG moieties, which was more favorable for IBD targeting compared to anionic and cationic surfactants (Lautenschlager et al., 2013).

### *In vitro* release study of PCur conjugate

**Figure 3.**
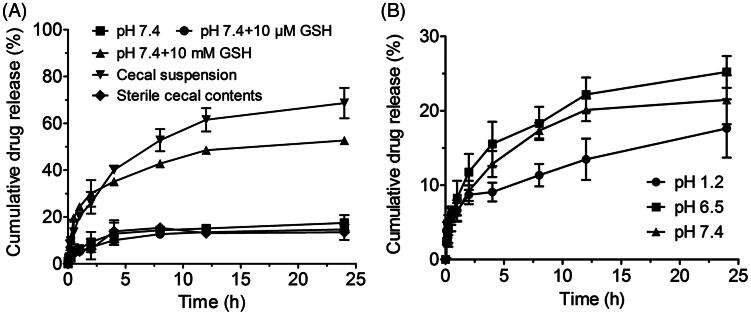
Reduction triggered Cur release from PCur conjugate in the presence of GSH and cecal suspension (A); *in vitro* release profiles of PCur conjugate under different pH conditions (pH 7.4, 6.5 and 1.2) (B); all data represent as mean ± SD (*n* = 3).

The *in vitro* release of PCur was performed in different media. GSH was used at the concentration of 10 μM or 10 mM to stimulate *in vitro* reduction level of extracellular and intracellular environment, respectively (Li et al., 2012). As shown in Figure 3(A), 10 μM GSH barely affected the slow and limited release of Cur from PCur in pH 7.4 buffer. For example, less than 10% of Cur was released in the first 2 h and approximately 15% within 24 h. However, with the presence of 10 mM GSH, the release of Cur was dramatically accelerated. Nearly 30% of Cur was released in 2 h and in total 53% within 24 h, which strongly supported our designing idea of using GSH concentration gradient to realize the redox-activated release of Cur from PCur.

The release was also evaluated *ex vivo* in a suspension of rat cecal content as an artificial model of the colonic environment, in which most molecules will get reduced to a similar extent as the human colon (Saphier et al., 2012). An accelerated release profile of Cur from PCur was discovered after the incubation with rat cecal suspension, which allows rough 70% of cumulative drug release within 24 h. However, it was noteworthy that the release of Cur in the control group of incubating with sterile cecal content was significantly slower and similar to that of PCur in the absence of GSH and even with 10 μM GSH. These phenomena indicated that PCur was inclined to decompose and subsequently release active Cur in a reducing environment such as intestinal canal, a desirable benefit presumably coming from the redox-sensitive disulfide bond incorporated in the polymer chain.

Next, the effect of solution acidity on the *in vitro* release of PCur was evaluated in buffers with pH value of 7.4, 6.5 and 1.2 to simulate different regions along the GIT. As shown in Figure 3(B), a consistent slow release of Cur was observed under all of the conditions. For example, only 10% of Cur was released within the first 2 h in buffer of pH 6.5, and less than 25% of Cur was freed even for 24 h. No significant pH-responsive release characteristic was witnessed during the predetermined period, indicating the robustness of PCur in environments with various pH values ranging from highly acidic stomach to slightly alkaline small intestine and the final destination of slightly acidic colon.

### *In vitro* stability testing of PCur conjugate

**Figure 4.**
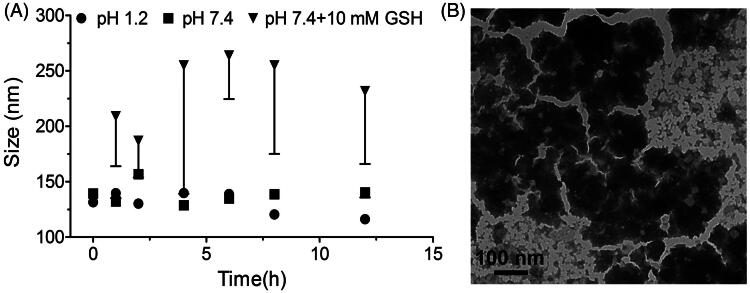
Real-time changes of PCur conjugate in size under pH 1.2 and 7.4 in absence/presence of 10 mM GSH (A); the morphology of PCur conjugate examined by TEM after incubation with 10 mM GSH for 12 h (B).

As shown in Figure 4(A), particle size distribution of PCur had little change in both pH 1.2 and 7.4 PBS at 37 °C, implying the structural integrity of PCur under different pH in the GIT. However, the presence of 10 mM GSH substantially increased the mean diameter of PCur accompanied with extended particle distribution. Indeed, as shown in TEM (Figure 4B), the particle experienced collapse and the original morphology had been completely disrupted. This result could be attributed to the breakage of disulfide bond of PCur responding to the highly reducing environment, leading to the destruction of nanoparticle and release of the active drug.

### Cytotoxicity study and LDH release assay

**Figure 5.**
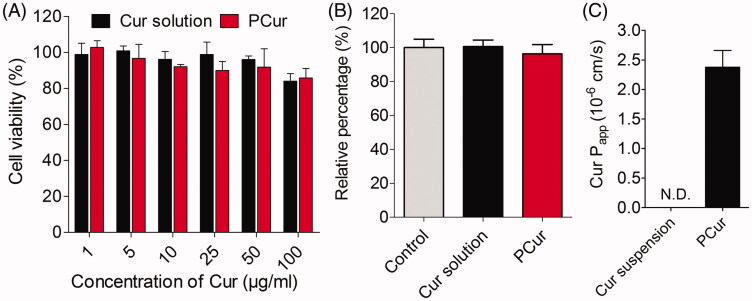
*In vitro* cytotoxicity (A) and LDH release assays (B) of Caco-2 cells after 2 h incubation with Cur solution and PCur, respectively; the apparent permeability coefficient (*P*_app_) of Cur suspension and PCur against Caco-2 cells (C).

The cytotoxicity of Cur and PCur were assayed on Caco-2 cells by MTT method. As shown in Figure 5(A), neither Cur solution nor PCur induced any significant changes in cellular viability at the concentrations of Cur from 1 to 100 μg/mL after incubation for 2 h, indicating that the general safety of Cur solution and PCur on Caco-2 cells. Meanwhile, compared with the control group, no significant LDH leakage from Caco-2 cells was determined with the addition of Cur solution or PCur at the concentration of 25 μg/mL Cur within 2 h (Figure 5B). Therefore, PCur was concluded to be incapable of breaking the integrity of the Caco-2 cellular membrane in such concentration.

### Transcellular studies

To estimate the transmembrane capacity of PCur, Caco-2 cell monolayer was built on the Transwell® and apparent permeability coefficient (*P*_app_) was determined to predict the permeability of Cur across the intestinal barrier. As the results showed in Figure 5(C), the *P*_app_ value of PCur conjugate was (2.38 ± 0.29) × 10^−6^ cm/s, while the amount of Cur in the BL compartment was too low to be detected for Cur suspension. Crohn’s disease, as one type of IBD and characterized by transmural inflammation, could affect the whole bowel wall and reach the serosal layer (Abraham & Cho, 2009). The poor transmembrane ability of Cur suspension may account for a limited and unsatisfactory inhibitory efficacy of Cur for IBD in clinical trials, which targeted preferentially intestinal epithelial cells rather than the deep focus (Lahiff & Moss, 2011).

As previously reported, PEG could increase the transmembrane capacity of Cur through either chemical conjugation or physical suspension (Zhongfa et al., 2012). On the basis of prior release study, transmembrane Cur could probably exist in both free and conjugated forms. In any manner, both forms will benefit from PEG with enhanced permeability and preferable drug distribution in the BL and the AP side of intestinal epithelia. It also enabled Cur with multi-pronged regulation and intervention to target different signaling pathway and transcription factors associated with IBD (Sreedhar et al., 2016).

### Pharmacokinetic study

**Figure 6.**
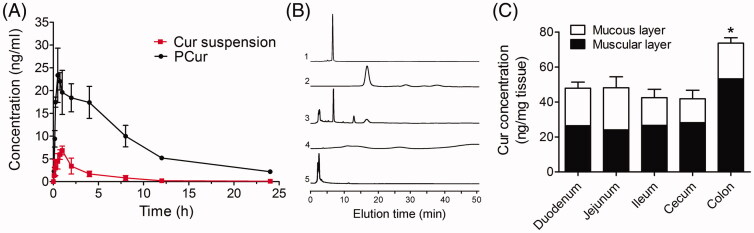
(A) Plasma concentration–time curves of Cur suspension and PCur after oral administration at the dose of 50 mg/kg Cur (*n* = 6); (B) HPLC analysis: (1) Cur (5 μg/mL), (2) PCur (100 μg/mL), (3) intestinal perfusate from mice receiving PCur, (4) PEG, (5) blank intestinal perfusate from mice receiving PBS; (C) quantitative assay of Cur in mucous and muscular layers from different intestinal segments, *p < 0.05 versus other groups.

Sprague–Dawley rats were used to study pharmacokinetics of orally administrated PCur and Cur suspension. Plasma concentration of Cur was determined by UPLC–MS/MS. As shown in Figure 6(A) and Table 1, the concentration–time profile of PCur was notably higher than that of Cur suspension. The *T*_1/2_ of PCur was 1.74-fold higher than Cur suspension, which emphasized the fact that PCur could extend the *in vivo* retention time of Cur. *T*_max_ of Cur suspension was 1.00 ± 0.02 h compared with shortened value of 0.50 ± 0.25 h for PCur, which is possibly a result of the rapid absorption of PCur after oral administration. 3.41-fold higher *C*_max_ of Cur was observed for PCur (23.35 ± 0.96 ng/mL) compared with Cur suspension (6.85 ± 0.36 ng/mL) after oral administration. In addition, the AUC_(0 − 24)_ value was increased 8.63 times from 23.42 ± 2.81 h ng/mL for Cur suspension to 202.05 ± 6.72 h ng/mL for PCur. Combining all the results, it can be concluded that PCur remarkably improved intestinal absorption and plasma drug concentration, which was attributed to elevated transcellular transport capacity referring to the above.

**Table 1.** Pharmacokinetic parameters of Cur after oral administration Cur suspension and PCur (50 mg/kg Cur, mean ± SD, *n* = 6).

Parameters	Cur suspension	PCur
*T*_1/2_ (h)	3.90 ± 1.25	6.77 ± 1.40
*T*_max_ (h)	1.00 ± 0.02	0.50 ± 0.25^a^
*C*_max_ (ng/mL)	6.85 ± 0.36	23.35 ± 0.96^b^
AUC_0–24__h_ (h ng/mL)	23.42 ± 2.81	202.05 ± 6.72^b^
AUC_0–∞_ (h ng/mL)	23.98 ± 4.89	223.52 ± 5.25^b^
MRT_0–∞_ (h)	4.71 ± 1.15	9.60 ± 2.28^a^

^a^*p* < 0.05.

^b^ *p* < 0.01 versus Cur suspension.

### *In vivo* distribution of PCur in intestinal tract of IBD mice

The stability of PCur to simulated gastrointestinal fluids and its ability to release Cur in response to reducing condition, motivated us to determine whether orally delivered PCur could target to inflamed intestinal tissues. Intestinal inflammation was induced in female C57BL/6 mice by replacing their drinking water on day zero with a 3% solution of DSS. Starting on day zero, mice receiving DSS were given a daily oral gavage of PCur (50 mg Cur per kg). On day seven, the intestinal perfusate and different intestinal segments were collected respectively for determining the biodistribution of the delivered Cur by HPLC. As shown in Figure 6(B), Cur and PCur exhibited characteristic peak at elution time of 7.2 and 17.6 min, respectively. Both PEG and blank intestinal perfusate showed no interference on Cur test. When perfusate from mice receiving PBS containing PCur was injected into HPLC, a new peak belonged to Cur appeared at the elution time of 7.2 min besides residuary PCur at 17.6 min, indicating efficient release of active Cur through cleavage of the disulfide linkage. Conversion for PCur was proposed that the disulfide bond involved in PCur was cleaved under a reducing condition to generate a sulfhydryl group; the resultant free thiol group would undergo an intramolecular nucleophilic acyl substitution on the ester moiety to release Cur molecule in its original active form (Yin et al., 2015).

Subsequently, mucous and muscular layers from different intestinal segments were measured for quantitation of Cur. As shown in Figure 6(C), Cur could preferentially accumulate in the colon, and the amount of Cur delivered to the colon showed a greater than 1.5-fold increase comparing with other intestinal segments (*p* < 0.05). Remarkably, Cur located in the mucous layers of colon displayed no significant difference comparing to other intestinal segments (*p* > 0.05), while proximately two-fold increase in the muscular layers. As the inflammation induced by DSS is mainly confined to the colon, these results confirmed that PCur could response to the bacterial reduction and target Cur to the inflamed colon tissues. Moreover, Cur preferentially delivered to the muscular layers of colon was probably attributed to the increased penetration of PCur and changed structure of intestinal wall resulted from inflammatory injury. Indeed, several events contribute to increased bacterial exposure in IBD, including disruption of the mucus layer, dysregulation of epithelial tight junctions, increased intestinal permeability and increased bacterial adherence to epithelial cells (Abraham & Cho, 2009). These factors probably led to extended reaction regions of PCur responsive to bacterial reduction, rather than limited in the intestinal lumen.

### Pharmacodynamics of PCur in a murine DSS-induced IBD model

#### Pathological analysis

**Figure 7.**
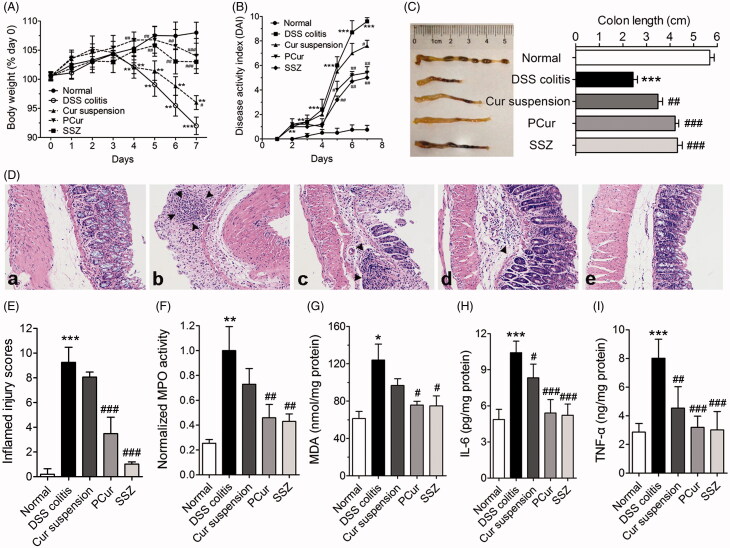
Orally administered PCur protected mice from DSS-induced IBD. Change in body weight (A), DAI evaluation (B) and colon length (C) of normal mice and DSS-induced mice receiving different treatments; (D) histological sections of colon from normal mice (a), DSS-induced mice (b), DSS-induced mice treated with Cur suspension (c), PCur (d) and SSZ (e) by stained with H&E; black arrow represented inflammatory infiltration; quantitative scores of inflamed degree (E); effects of PCur administration on MPO activity (F), MDA content (G), IL-6 (H) and TNF-α (I) levels in colonic tissues. *p < 0.05, **p < 0.01, ***p < 0.001 versus normal group, #*p* < 0.05, ##*p* < 0.01, ###*p* < 0.001 versus DSS-induced group.

We also investigated the efficacy of orally delivered PCur to allay the clinical manifestations of DSS-induced IBD. Overall, a strong protection of mice DSS-induced IBD from PCur was clearly validated based on the results from weight loss, DAI scores, the length of colon and histological analysis. As shown in Figure 7(A), less weight loss was observed in DSS-induced IBD mice with the treatment of PCur or SSZ than DSS or Cur suspension. Moreover, the DAI, a vital indicator of the severity of intestinal inflammation, was used to analyze the therapeutic efficacy of each treatment. Mice subjected to the oral administration of DSS regularly developed IBD with weight loss, severe diarrhea and rectal prolapse accompanied by extensive wasting disease, which were successfully mitigated to varying degree especially in PCur and SSZ group (Figure 7B). Furthermore, the shortened length of the mice colon, a typical symptom of DSS-induced IBD, was significantly recovered after the treatment of therapeutic agents, where PCur and SSZ group displayed the strongest efficacies (Figure 7C). Finally, histological examination was performed after H&E staining (Figure 7D and E). The colons of mice receiving DSS and treatment with PCur had intact epitheliums, well-defined crypt structures and relatively low levels of neutrophil invasion in comparison with that of DSS-induced mice receiving PBS or Cur suspension treatment.

#### Determination of biomarker and cytokine

A spectrum of biomarkers and cytokines associated with IBD were assessed to estimate the therapeutic efficacy. As shown in Figure 7(F,G), mice with DSS-induced IBD had significantly elevated MPO activity and MDA content, which were strongly decreased after oral administration of PCur with a similar inhibition effect to the positive group of SZZ (*p* > 0.05). However, no significant alleviation of IBD was detected in mice with Cur suspension treatment, which is probably attributed to the nonspecific gastrointestinal distribution and the poor oral bioavailability of Cur suspension.

DSS-induced IBD was accompanied by the release of pro-inflammatory cytokines like IL-6 and TNF-α, leading to numerous inflammatory infiltration and dysfunction. As shown in Figure 7(H,I), the increase of IL-6 and TNF-α expression was significantly reduced after the treatment of agents in mice with DSS-induced colitis (*p* < 0.05–0.001). It was found that the group with treatment of PCur or SSZ displayed the strongest efficacy in the inhibition of the production of IL-6 and TNF-α induced by DSS, and the therapeutic effects were comparable to each other (*p* > 0.05). Less inhibitory efficacy measured in Cur suspension (*p* < 0.01 versus PCur or SSZ) was possibly a result of nonspecific release and poor epithelial penetration resulting in limited regulation of inflammatory cytokines.

Above all, orally administrated PCur could significantly alleviate the DSS-induced symptom in mice evidenced by multi-pronged regulation and intervention in a number of indicators relative to IBD. There are several reasons that could account for the increased therapeutic efficacy. First, PCur can increase the solubility and partition coefficient of Cur, facilitating the transmural distribution to reach the BL side even the serosal layer, where a wide range of inflammatory infiltration and dysfunction are involved (Abraham & Cho, 2009). Moreover, transportation across intestinal epithelia is essential for the drug to enter the systemic circulation. As already stated above, pharmacokinetic results confirmed the elevated plasma drug concentration and improved bioavailability for mice receiving orally administrated PCur. Circulating nano-scaled drug could target inflammatory regions from the backside through the blood-endothelial pathway as a result of the well-established enhanced permeability and retention effect (EPR) and the uptake by the infiltrated inflammatory cells (Youshia & Lamprecht, 2016). Undoubtedly, this cascading consequence would further enhance the inflammation-specific therapeutic efficacy of PCur for IBD injury.

## Conclusion

Oral administration remains the most convenient and cost-effective means to deliver therapeutic agents to diseased intestinal tissues, while a wave of factors such as gastrointestinal fluids, the intestinal mucosa and cellular barriers to uptake still impede the success of orally delivered drugs. We hereby reported the fabrication of an amphiphilic PCur conjugate containing disulfide bond to act responsively to the intestinal bacterial reduction environment. The results from both *in vitro* and *in vivo* highlighted the fact that PCur possessed the needed chemical and physical properties to overcome these obstacles and provide a significantly higher therapeutic level of Cur in inflamed intestinal tissues. Taken together, it can be expected that PCur will make a more crucial contribution to the treatment of colon-related inflammatory diseases.
